# Systematic Review on Diagnostic Reference Levels for Computed Tomography Examinations in Radiation Therapy Planning

**DOI:** 10.3390/diagnostics13061072

**Published:** 2023-03-11

**Authors:** Shreekripa Rao, Krishna Sharan, Suresh Sukumar, Srinidhi Gururajarao Chandraguthi, Rechal Nisha Dsouza, Leena R. David, Sneha Ravichandran, Berna Uzun, Rajagopal Kadavigere, Dilber Uzun Ozsahin

**Affiliations:** 1 Department of Radiotherapy and Oncology, Manipal College of Health Professions, Manipal 576104, Karnataka, India; 2 Department of Radiotherapy and Oncology, Kasturba Medical College and Hospital, Manipal 576104, Karnataka, India; 3 Department of Medical Imaging Technology, Manipal College of Health Professions, Manipal 576104, Karnataka, India; 4 Department of Medical Diagnostic Imaging, College of Health Sciences, University of Sharjah, Sharjah 27272, United Arab Emirates; 5 Department of Statistics, Carlos III Madrid University, 28903 Madrid, Spain; 6 Operational Research Centre in Healthcare, Near East University, TRNC Mersin 10, Nicosia 99138, Turkey; 7 Department of Mathematics, Near East University, TRNC Mersin 10, Nicosia 99138, Turkey; 8 Department of Radiodiagnosis and Imaging, Kasturba Medical College and Hospital, Manipal 576104, Karnataka, India

**Keywords:** radiotherapy, diagnostic reference levels, head and neck, pelvis, DLP, CTDIvol

## Abstract

**Background**: In August 2017, the European Commission awarded the “European Study on Clinical Diagnostic Reference Levels (DRL) for X-ray Medical Imaging” project to the European Society of Radiology to provide up-to-date Diagnostic Reference Levels based on clinical indications. This work aimed to conduct an extensive literature review by analyzing the most recent studies published and the data provided by the National Competent Authorities to understand the current situation regarding Diagnostic Reference Levels based on clinical indications for Radiation Therapy Computed Tomography. **Objective**: To review the literature on established DRLs and methodologies for establishing Diagnostic reference levels in radiation therapy planning computed tomography (RTCT). **Methods**: Eligibility criteria: A cohort study (observational design) reporting DRLs in adult patients undergoing computed tomography (CT) for radiation therapy for the region head and neck or pelvis were included. The comprehensive literature searches for the relevant studies published between 2000 and 2021 were performed using PubMed, Scopus, CINHAL, Web of Science, and ProQuest. **Results**: Three hundred fifty-six articles were identified through an extensive literature search. Sixty-eight duplicate reports were removed. The title and abstract of 288 studies were assessed and excluded if they did not meet the inclusion criteria. Sixteen of 288 articles were selected for full-text screening (studies conducted between 2000 and 2021). Five articles were included in the review after the full-text screening. **Conclusions**: A globally approved standard protocol that includes scanning techniques, dose measurement method, and DRL percentile needs to be established to make a valuable and accurate comparison with international DRLs.

## 1. Introduction

A cancer patient might go through multiple computed tomography (CT) examinations from diagnosis to treatment [1]. High quality CT data are more in demand as a result of a number of sophisticated radiation oncology methods [2]. The widespread use of multi-detector computed tomography (MDCT) has led to a significant increase in the number of CT scans performed annually. This is due to a number of factors, including the high level of detail that can be obtained from MDCT scans, the speed at which the scans can be performed, and the increasing availability of MDCT machines. However, this increase in the use of CT scans has raised concerns about the potential radiation exposure associated with the procedure, as well as the associated costs.

As a result, there has been increased focus on developing guidelines and protocols to ensure that CT scans are used judiciously and that patients are exposed to the lowest possible radiation dose. This includes the development of dose reduction techniques, such as iterative reconstruction algorithms, as well as efforts to optimize the use of CT scans through the implementation of decision support tools and the use of alternative imaging modalities when appropriate [3].

Radiation-induced cancers and fatalities are linked to the use of ionizing radiation on individuals [4]. The study mentioned, along with other pieces of literature, suggests that children and adolescents who undergo CT scans are at a statistically significant increased risk of developing malignancy [5,6]. Additionally, radiation-induced damage to deoxyribonucleic acid (DNA) increased and it is documented [7].

Radiation therapy is typically planned using 3D CT images of the patient obtained from a CT simulator. These images help to identify the position of the tumor relative to anatomical landmarks or fiducial markers. To ensure accurate delivery of the radiation dose, imaging is also performed during treatment to confirm the patient’s position [8,9,10].

CT images taken for radiation oncology purposes typically require an extended scanning area to encompass the entire treatment area, which can result in an increased absorbed dose of radiation to the patient. This is because the larger the scanning area, the more tissue that is being exposed to ionizing radiation, leading to a higher accumulated dose.

It is important to consider the potential risks associated with radiation exposure and weigh them against the potential benefits of the imaging examination. In many cases, the information obtained from the CT scan is essential for planning effective radiation therapy and ensuring that the right dose of radiation is delivered to the right location. However, in some cases, alternative imaging methods that use lower levels of radiation, such as ultrasound or magnetic resonance imaging (MRI), may be used instead.

4D radiation delivery, also known as four-dimensional conformal radiation therapy, involves capturing multiple images of the same anatomy over several respiratory cycles in order to plan and deliver the most precise and effective radiation treatment possible. However, acquiring these images requires multiple CT scans, which can result in a significant increase in the total absorbed dose due to ionizing radiation exposure. This highlights the importance of carefully considering the risks and benefits of 4D radiation delivery and choosing the most appropriate radiation dose protocol for each individual patient. Additionally, efforts are being made to optimize CT imaging protocols and reduce radiation doses in order to minimize the potential risks associated with 4D radiation delivery [2].

Swift advances in multiphase multi-detector computed tomography have made possible the multiplanar reconstruction of isotropic images. Multiple detector arrays enable high-speed acquisition of isotropic photos, and image reconstruction is rapid. A crucial aspect of radiation treatment is computed tomography (CT) [11]. Obtaining digitally reconstructed radiographs for setup verification of the patient on the teletherapy unit before starting the treatment also makes it easier to deliver radiation accurately. Despite these benefits, a major disadvantage of computed tomography is the use of ionizing radiation for imaging, specifically the possibility of stochastic consequences. According to the ALARA principle, imaging dosages given to patients should be as low as is practically possible.

The fundamental CT dose quantities in the CT control console are the dose-length product (DLP) and the volumetric CT dose index (CTDIvol). The aforementioned measurements are not direct patient doses, but they are related to patient exposure and serve as an important criterion for the technical assessment of different CT systems. Numerous articles have assessed patient exposure levels during diagnostic computed tomography, and a diagnostic reference level is provided [1].

The diagnostic reference level is the dose level for standard-sized patients undergoing routine examinations in diagnostic radiography practice for a broadly defined equipment group. A diagnostic reference level is a type of investigation level that is used as a tool to help ensure that patients are protected as much as possible when they are exposed to medical treatments for both diagnostic and interventional procedures [11].

Various radiological procedures constitute different Diagnostic Reference Levels (DRL) implemented by the International Council of Radiation Protection (ICRP). The ICRP encourages the establishment of a standard DRL to represent a particular medical practice in a specific geographical area. DRLs will not differentiate between fair and unfair medical practices. The main objective of the suggested DRL is to minimize the radiation dose and improve the image quality of a medical practitioner [11,12,13,14].

DRLs are not applicable in radiation practice. However, it is vital to use DRLs for various imaging techniques in radiation therapy treatment, imaging to verify the patient’s position, and treatment planning. The system of diagnostic reference levels was formulated as a tool to aid in dose optimization for diagnostic and interventional medical imaging procedures. In 1996, the International Commission of Radiation Protection (ICRP) introduced DRLs as a quality control method. DRLs aim to validate dose optimization strategies to avoid unnecessary patient exposures to radiation while maintaining the diagnostic integrity of the medical imaging task. DRLs are a form of investigation level applied to an easily measured quantity, usually the absorbed dose in the air or tissue-equivalent material at the surface of a simple standard phantom or a representative patient.

The IAEA and WHO provide recommendations for diagnostic reference levels (DRLs) in radiology as guidelines for safe and efficient use of medical radiation. These DRLs serve as a benchmark for the amount of radiation that should be used in different imaging procedures and help ensure that patients receive the minimum amount of radiation necessary for accurate diagnosis, while also reducing the risk of harm from unnecessary exposure [15,16,17,18].

Diagnostic reference levels are an essential tool used by radiology departments for minimizing radiation doses, and regulations for their use have been established in many countries. However, several limitations in the methods used for selecting these National DRLs (NDRL) have been extensively addressed in several studies. The different Multi-detector Computed Tomography (MDCT) systems included in most published NDRLs studies significantly affect DRL values [19,20,21].

## 2. Survey Methodology

The current review protocol was registered and approved by PROSPERO (CRD4202188381).

### 2.1. Keyword Builds and Search Strategy

Using the Cochrane library, Medical term (MeSH) search engine, all necessary terms for the keywords “Diagnostic Reference Level” along with “Radiation Therapy” were identified, and the search string was built using appropriate Boolean operators. (Keywords: “Reference level *’’ OR “Diagnostic reference levels” AND “CT scan” OR “Computed Tomography” OR “CAT scan” OR “computed axial tomography” AND “Radiotherapy Dosage” OR “Radiotherapy” The comprehensive literature searches for the relevant studies published during 2000–2021 were performed using PubMed, Scopus, CINHAL, Web of Science, and ProQuest. The method of study retrieval for each is reported in (Table 1) The search was predominantly run through the title and abstract of all articles published until October 2021.

### 2.2. Type of Study

The original studies published in English between 1975 and 2021 were observational cross-sectional studies that reported the Diagnostic Reference Levels in Radiation therapy CT planning.

### 2.3. Data Collection and Analysis

The articles for inclusion in this review were selected after employing the search strategy mentioned in Table 2. All the selected papers were uploaded in the Rayyan tool to identify the duplicates and screen title and abstract, and five articles were identified.

### 2.4. Removing Duplicates

Each study was scrutinized to ensure that multiple publications of the same study were included only once. All articles’ duplicates were removed and linked to a single study.

### 2.5. Level 1: Article Screening

The first level of article screening was examination based on the article title only. Two blinded reviewers reviewed all resulting article titles. Articles that were unrelated to the research questions were removed at this level. We included those in the abstract review in case of doubts about the relevance of an article title.

### 2.6. Level 2: Abstract Review

Two independent reviewers reviewed the abstracts of all articles included from level 1. The third reviewer resolved conflicting results between the two reviewers. The decision of the third reviewer was considered final.

### 2.7. Level 3: Full-Text Review

All records selected from level 2 were reviewed using the full text. Two independent researchers examined the complete study of all articles from level 2. The two reviewers collated the results and reported the findings to the third reviewer. The third reviewer resolved the conflicting results between the two reviewers, and made the final decision.

### 2.8. Level 4: Reference List Search

The reference list of each selected article was thoroughly reviewed to ensure that no relevant articles were overlooked.

### 2.9. Data Extraction and Management

The authors extracted data from the included articles using an Excel spreadsheet with the following entries.

#### 2.9.1. General Information

Title of the article;

Main author;

Publication year;

Study-related data;

Study design;

Sample size.

#### 2.9.2. Participant-Related Data

Age (mean, SD; range);

Mean Dose Length Product (DLP), effective dose;

The tube voltage and tube current;

DLP.

### 2.10. Quality Assessment

Data from the articles were assessed for quality of methods and outcomes. The reviewers evaluated the quality of the articles based on an assessment tool for quantitative studies developed by the Effective Public Health Practice Project. The articles were rated high [1] and moderate [2]. Two authors independently rated the articles; all the articles were rated moderate.

**Figure 1:**
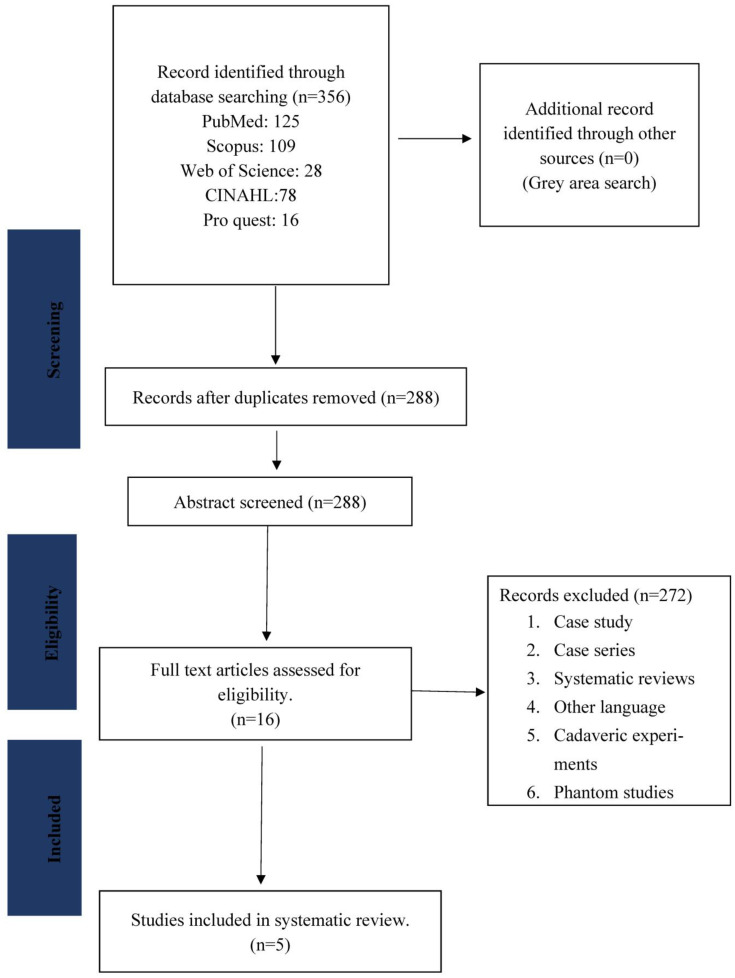
PRISMA Flow-Chart.

The PRISMA flow chart (Figure 1) provides a helpful and straightforward way of documenting the selection of articles at each stage of the screening process [22].

## 3. Results

Three hundred fifty-six articles were identified through an extensive literature search. Sixty-eight duplicate reports were removed. The title and abstract of 288 studies were assessed and excluded as they did not meet the inclusion criteria. Next, 16 of 288 articles were selected for full-text screening (studies conducted between 2000 and 2021). Five articles were included in the review after the full-text screening. All the above processes were completed via Rayyan QCRI [23].

## 4. Characteristics of Included Studies

- Type of the study design (phantom, human, or both).
- Whether DRLs were established on third quartile or mean.
- Countries are indicating where the studies were conducted.
- Sample size reported in several scanners or patients or hospitals.
- DRLs in terms of radiation dose indices: volume computed tomography dose index (CTDI_vol)_, weighted computed tomography dose index (CTDI_w_), dose length product (DLP), effective dose (ED), specific size dose estimate (SSDE).
- Whether the articles reported that the scanners had undergone prior quality control (QC) tests.

Characteristics of the five included studies are tabulated in Table 3.

DRLs for Head and Neck, Breast, Thorax, and Abdomen based on CTDI_vol_ and DLP only are tabulated in Table 4.

DRLs for angiography for Coronary CT angiogram based on CTDI_vol and_ DLP are tabulated in Table 5.

**Figure 2:**
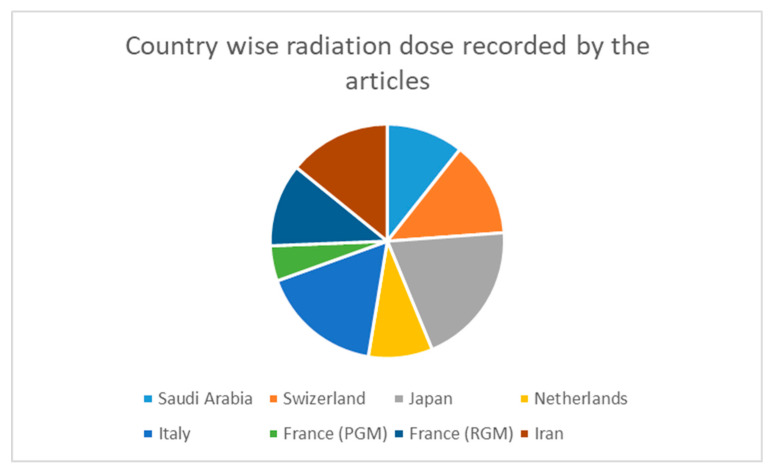
CCTA DRLs in Saudi Arabia compared with other international DRLs.

CCTA DRLs in Saudi Arabia compared with other international DRLs are tabulated in Table 6 and Figure 2.

**Figure 3:**
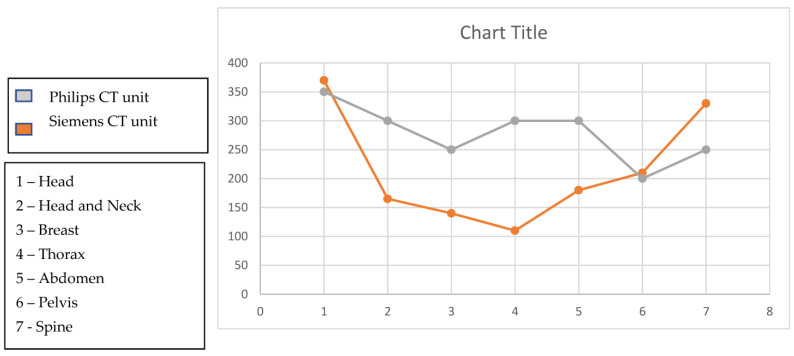
Comparison of mAs between Siemens CT unit and Philips CT unit.

Scanning parameters of the simulation protocols in the Siemens CT unit and Philips CT unit are tabulated in Table 7 and Table 8 respectively, a combined comparison is given in Figure 3.

## 5. Discussion

As reported in the literature, the diagnostic reference levels (DRLs) are values that serve as a reference point for comparing the radiation dose in medical imaging exams, such as computed tomography (CT) scans. The purpose of a DRL is to provide a benchmark for ensuring that radiation doses in medical imaging are as low as reasonably achievable (ALARA) while still producing the necessary diagnostic information.

Several studies have reviewed the methodologies used to establish DRLs for radiotherapy CT scans. These studies have evaluated different methods for calculating DRLs, including statistical analysis of data from a large number of exams and the use of expert opinion. The results of these studies have shown that different methods can lead to different DRL values, and the choice of methodology can impact the final DRL value.

It is important to note that while DRLs provide a useful benchmark, they should not be used as an absolute standard, as the appropriate radiation dose for an individual patient will depend on many factors, including their medical history, the type of exam being performed, and the specific imaging equipment being used [22].

The utilization of diagnostic reference levels as an essential dose optimization tool is endorsed by many professional and regulatory organizations, including ICRP, American College of Radiology (ACR) and American Association of Physicists in Medicine (AAPM). Reference levels are typically set at the 75th percentile of the dose distribution from the survey conducted across a broad user [2]. In recent years, there has been growing concern about the potential risks associated with exposure to ionizing radiation from medical imaging procedures, particularly from repeated CT scans. To address this issue, many CT vendors have developed technologies and techniques to help optimize the radiation dose in patients who are undergoing multiple scans.

One of these measures is Automated exposure control (AEC). This technology adjusts the radiation dose based on the patient’s size and anatomy, helping to ensure that the right amount of radiation is used for each individual. Another measure is Adaptive Statistical Iterative Reconstruction (ASIR), which uses mathematical algorithms to reduce the amount of radiation needed to produce a diagnostic image. By reducing the amount of radiation that is emitted during a CT scan, the total radiation dose can be decreased, while still producing high-quality images. Finally, one more technique is Image gating. This is a technique that uses specialized software to limit the amount of radiation that is delivered to the patient during specific phases of the scan.

Hospitals and imaging centers have also implemented protocols and guidelines to ensure that patients are only subjected to the minimum amount of radiation necessary to produce a diagnostic image. These include using alternative imaging modalities whenever possible and limiting the number of unnecessary scans.

Overall, these efforts are helping to minimize the radiation exposure for patients and reduce the potential for long-term health effects associated with repeated exposure to ionizing radiation [28]. According to an ICRP document, surveys on patient doses must be conducted in order to determine DRLs internationally for imaging modalities. Since the patients referred for computed tomography in radiotherapy are the patients who undergo the scan multiple times in a short period, it is essential to reduce the unwanted radiation dose that has been delivered to the patients that may cause radiation-induced cancer.

There are various techniques to reduce radiation; one is establishing the standard diagnostic reference level for multiple procedures in respective modalities. The technical advances to reduce radiation dose include iterative reconstruction techniques, tube voltage, current modulation, and Bow tie filters. In total, in this review five articles met our inclusion criteria. Various studies have used different dose indices for establishing DRLs. Most of the studies [13,24,25,27] reported CTDI_vol_ index over CTDI_w_ since most of the scanners were multi-slice and programmed in a spiral model; this incorporates pitch in, the quantification of the dose output for a specific protocol, whereby the dose index is represented by CTDI_vol_ [22]. Overall, CTDI_vol_ and DLP are the recommended dose indices for CT DRLs by ICRP, and the European commissions are the most widely reported dose metrics found in the current review [22]. Even though CTDI_vol_ and CTDI_w_ are still being used to register DRLs in various studies, comparison of doses is a challenge because these two dose indices are not the same. CTDI_vol_ is a derivative of CTDI_w_, but with the inclusion of the pitch value [22] Only one study [26] in 2018 reported CTDI_vol_ and DLP of 21 mGy and 88 2mGy.cm, respectively, for establishing DRLs for head and neck, while another study by Toroi et al. [26] reported a CTDI_vol_ for head and neck 29 mGy comparatively higher than another study. The articles reviewed by Toroi et al. [26] are the only studies reporting CTDI_vol_ as DRLs (Table 4). The comparison may be made with neck DRLs without RT-specific data. An Irish study reported a national DRL for the C-spine of 420 mGycm and 19 mGy, and published EU data for the C-spine of 460 mGycm and 19 mGy. Australian national data for neck CT were reported to be 600 mGycm and 30 mGy, and the USA recorded from 13 centers a DRL of 650 mGycm and 23 mGycm [24]. Sanderud et al. [29] suggested that the lack of patient body mass index was the main reason for higher patient dose in the RT setting compared to DI. The quality assurance procedures and the accuracy of the CT machine DLP and CTDI_vol_ outputs were not assessed [24]. A study by Alhaily et al. [27] used a mixed model, prospective and retrospective ECG gating mode for coronary angiogram, CTDI_vol_ were 43 and 27 for the hybrid model in 75th and 25th percentile respectively (Table 6). DLP was 808 and 359 for the 75th and 25th percentile for the same.

DRLs in Saudi Arabia (2018) were comparatively less (CTDI_vol_ 43, DLP 808) compared to international DRLs; international DRLs were like following, Iran 66.5 mGy, 1073mGycm being the highest for Coronary angiography computed tomogram, and Switzerland (2010) with CTDI_vol_ of 50, DLP of 1000 (Table 6). Usually, scanning parameters for CCTA are adapted based on body weight and BMI. The mean patient weight for most studies ranges from 75 to 90 k. The scan canters were not adjusted based on the patient indication in many centers. The Z-axis coverage examination is the main factor for the increase or decrease in the dose indices. Numerous studies have been performed on various scanners like GE, Philips, and Siemens.

Scanning protocols in Siemens CT unit for Head, Head, and Neck, Breast, Thorax, Abdomen, Pelvis, spine has almost similar scanning parameters including acquisition type, voltage (kV) reference (mAs), collimation being 20 × 0.6 for head scan and 16 × 1.1. Automatic exposure control was on for all the above scans, and rotation time and pitch differed slightly (Table 7). Similarly, Philips CT unit varied significantly (Table 8).

The American Association of Physicists in Medicine (AAPM) recommends reviewing the CT scan protocols annually [30]. Diagnostic reference levels (DRLs) provide guidance on the typical radiation doses used in medical imaging procedures, but they should not be used as a strict limit or goal. Instead, they serve as a reference point for optimization efforts, and the goal of dose optimization should always be to achieve the lowest possible dose while still maintaining the diagnostic quality of the images. This means that, in some cases, it may be necessary to use higher doses in order to obtain the information required for a diagnosis, and in such cases, the diagnostic benefits should take priority over concerns about radiation exposure. It is important to keep in mind that DRLs are not one-size-fits-all and may vary based on factors such as patient size, imaging technique, and equipment [25].

One study found a massive amount of variation in the exposure parameters used for the CT simulations. The default setting recommended by the manufacturer often selects these exposure parameters. Additional and typical optimization is typically not performed by the manufacturer, and continuous optimizations are not performed for these CT systems. The board questioned the justification for the higher dose levels required for diagnostic imaging in the annual meeting with radiation therapy physicists. CT simulation is the only part of the diagnostic and therapy process; therefore, other related exposures should also be evaluated [26].

In future studies, we recommend including studies using artificial intelligence (AI) to calculate the DRL. Integrating AI into medical imaging has brought about significant changes in radiology, enabling faster and more accurate diagnosis and treatment planning. AI algorithms are being developed and trained to identify patterns and features in medical images, such as X-rays, CT scans, and MRI scans, indicative of various diseases, enabling earlier and more accurate diagnoses. There are many studies related to artificial intelligence in medicine including the articles “AI-Assisted Tuberculosis Detection”, “Classification of Malaria Using Object Detection Models”, “Deep Learning Classifier for Detection of Acute Lymphoblastic Leukemia Using Blood Smear Images”, “Uses of AI in Ultrasound Imaging”, and “Machine Learning in Prostate MRI for Prostate Cancer” [31,32,33,34,35]. The integration of AI into medical imaging is an exciting development that has brought about many changes. AI can be a powerful tool to improve patient outcomes, but it must be used in conjunction with the expertise and judgment of a trained radiologist. In this study, we included mostly studies from European countries and a few from Asian Countries.

## 6. Conclusions

The commonly used method of collecting data to establish DRLs, as indicated in the ICRP and European commission documents, was based on all patients visiting the centers for RTCT examinations. Wide variations in CT doses for the same procedure of up to a factor of 2 were noted in phantom studies [22]. Radiographers play a crucial role in reducing radiation exposure to patients during medical imaging procedures. They can minimize the dose by using appropriate techniques, such as selecting the appropriate imaging modality, optimizing image quality, and using shielding where necessary. However, this can only be achieved if they have received proper education and training on dose-minimizing techniques. By increasing radiographers’ awareness of these techniques, they can consistently apply them in their daily work, leading to a significant reduction in variations in dose reference levels (DRLs). This, in turn, helps to ensure patient safety and reduce the risk of long-term harm from radiation exposure [36]. A globally approved standard protocol that includes scanning techniques, dose measurement methods, and a DRL percentile needs to be established to make a valuable and accurate comparison with international DRLs. Future studies should use age, weight band, and BMI as recommended by ICRP135 and European guidelines [28].

## Figures and Tables

**Table 1:** Database search.

Database	Number of Studies	Total
PubMed	125	356
Scopus	109	
Web of Science	28	
FINAL	78	
ProQuest	16	

**Table 2:** Keywords for literature search.

Sl. No.	Search
1	“Reference level” OR “Diagnostic reference levels”
2	“CT scan” OR “Computed Tomography” OR “CAT scan” OR “computed axial tomography”
3	“Radiotherapy Dosage” OR “Radiotherapy”

**Table 3:** Characteristics of the five included studies.

No.	Study	Year	Country	Design	Sample	Dose Indices	DRLs	QC	Quality Score
1	Celine et al. [24]	2018	Ireland	Prospective	25	CTDI_vol_ and DLP	75th percentile	Yes	M
2	Nika et al. [13]	2020	Slovenia	Retrospective	1631	CTDI_vol_ and DLP	3rd quartile	Yes	M
3	Sean et al. [25]	2016	Ireland	Prospective	60	CTDI_vol_ and DLP	3rd quartile	yes	M
4	Toroi et al. [26]	2014	Finland	Prospective	13 hospitals	CTDI_vol_	3rd quartile	Yes	M
5	Alhailiy et al. [27]	2018	Saudi Arabia	Retrospective	197	CTDI_vol_ and DLP	75, median and 25th percentile	Yes	M

CTDI_vol_—Computed tomography dose index, DLP—Dose length product.

**Table 4:** DRLs for Head and Neck, Breast, Thorax, and Abdomen based on CTDI_vol_ and DLP only.

Articles	Year	CTDI_vol_ (mGy)				DLP (mGy.cm)			
		Head and Neck	Breast	Thorax	Abdomen	Head and Neck	Breast	Thorax	Abdomen
Celine et al. [24]	2018	21	----	----	----	882m	----	----	----
Nika [13]	2020	(n = 278) 16.9	(n = 298) 11.2	(n = 289) 19.2	(n = 128) 18.2	(n = 278) 969.2	(n = 298) 606.6	(n = 289) 832.4	(n = 128) 1116.2
Sean et al. [25]	2016	----	26	----	----	----	732	----	----
Toroi et al. [26]	2014	29	24	13 (whole brain)	----	----	----	-----	----

CTDI_vol_—Computed tomography dose index, DLP—Dose length product.

**Table 5:** DRLs for angiography for Coronary CT angiogram based on CTDI_vol and_ DLP.

Article	Year	Scan Type	CTDI_vol_			DLP		
			75th	Median	25th	75th	Median	25th
Alhailiy et al. [27]	2018	Mixed models	43	37	27	808	554.5	359
		PGM	29	24	19	393	343	313
		RGM	62	46	40	1057	808	605
		CS test	5.8	4	3.7	69	8	46

CS—calcium score; CTDI_vol_—Computed tomography dose index, DLP—Dose length product. PGM—Prospective ECG gating mode; RGM—Retrospective ECG gating mode

**Table 6:** CCTA DRLs in Saudi Arabia compared with other international DRLs.

Study	CTDI_vol (mGy)_	DLP (mGy.cm)	CS (mGy.cm)
Saudi Arabia	43	808	69
Switzerland (2010)	50	1000	150
Japan (2012)	-	1510	-
Netherlands (2013)	-	671	-
Italy (2014)	61	1280	131
France (2014)	(PGM) 26 (RGM) 44	370 870	-
			-
Iran (2016)	66.5	1073	187

**Table 7:** Scanning parameters of the simulation protocols in Siemens CT unit.

Siemens	Acquisition Type	Voltage (kV)	Reference (mAs)	Collimation (n × mm)	AEC	Rotation Time	Pitch	Slice Thickness	Kernel
HEAD	Helical	120	370	20 × 0.6	On	1	0.55	3	H30s
Head and neck	Helical	120	165	16 × 1.1	On	1	0.8	2	B31s
Breast	Helical	120	140	16 × 1.2	On	0.5	1	3	B31f
Thorax	Helical	120	110	16 × 1.2	On	0.5	1.2	3	B31f
Abdomen	Helical	120	180	16 × 1.2	On	0.5	0.6	3	B30f
Pelvis	Helical	120	210	16 × 1.2	On	0.5	0.8	3	B30f
Spine	Helical	120	330	16 × 1.2	On	1	0.8	3	B30s

AEC—Automatic Exposure Control.

**Table 8:** Scanning parameters of the simulation protocols in Philips CT unit.

Siemens	Acquisition Type	Voltage (kV)	Reference (mAs)	Collimation (N × mm)	AEC	Rotation Time	Pitch	Slice Thickness	Kernel
HEAD	Helical	120	350	16 × 1.5	Off	0.75	0.93	3	UB
Head and neck	Helical	120	300	16 × 0.75	On	1	0.94	2	A
Breast	Helical	120	250	16 × 1.5	On	0.75	0.81	3	C
Thorax	Helical	120	300	16 × 1.5	On	0.75	0.81	3	B
Abdomen	Helical	120	300	16 × 1.5	On	0.75	0.81	3	B
Pelvis	Helical	120	200	16 × v1.5	On	0.75	0.68	3	A
Spine	Helical	120	250	16 × 1.5	On	1	0.98	3	A

AEC—Automatic Exposure Control.

## Data Availability

It is available within the manuscript.
